# The Effects of Six *Brassica napus* Cultivars on the Life Table Parameters of the Green Peach Aphid *Myzus persicae* (Sulzer) (Hemiptera: Aphididae)

**DOI:** 10.3390/insects16070726

**Published:** 2025-07-17

**Authors:** Mi Tian, Lin-Kui Li, Feng Zhu, Shi-Ze Zhang

**Affiliations:** 1 College of Life Science and Agri-Forestry, Southwest University of Science and Technology, Mianyang 621010, China; linkui_li@163.com (L.-K.L.); 15528675229@163.com (F.Z.); 2 Key Laboratory of Integrated Pest Management on Crops in Northwestern the Loess Plateau of Ministry of Agriculture and Rural Affairs, College of Plant Protection, Northwest A&F University, Yangling 712100, China

**Keywords:** *Myzus persicae*, oilseed rape, life table, Brassicaceae, host plant cultivars

## Abstract

The contents of glucosinolates and erucic acid clearly vary in *Brassica napus* seeds, but a few studies still focus on the effects of *B. napus* cultivars on the life table parameters of *Myzus persicae*. In this study, the life history parameters of *M. persicae* in six *B. napus* cultivars were examined at 25 ± 1 °C, 50 ± 10% RH, and a photoperiod of 14 h of light/10 h of dark under laboratory conditions. The results showed that significant differences exist in the life table parameters of *M. persicae* in six *B. napus* cultivars. The female fecundity, net reproductive rate, intrinsic rate of increase, finite rate of increase, and total longevity of *M. persicae* were higher in Xinong 18, Aiganyou 558, and Aiyouku 999. However, the intrinsic rate of increase in *M. persicae* was significantly lower in Zhongshuang 11 (r_m_ = 0.28 ± 0.006) and Mianxinyou 78 (r_m_ = 0.23 ± 0.007), suggesting the host-induced suppression of *M. persicae* population growth. Among all the cultivars mentioned above, Zhongshuang 11 and Mianxinyou 78 were recommended for planting for decreasing *M. persicae* population numbers. Additionally, more attention should be paid to Xinong 18, Aiyouku 999, and Aiganyou 558 to control *M. persicae* populations, and we conclude that *Brassica napus* cultivars affect the developmental duration and the population dynamics of *M. persicae*.

## Simple Summary

Oilseed rape, *Brassica napus* L., is the third-largest oil crop in the world, and it is susceptible to being infected by different pests, especially aphids. However, only a few studies have reported the effects of rapeseed cultivars on the life table of *Myzus persicae*. In this study, the life history parameters of *M. persicae* in six *B. napus* cultivars were analyzed by using an age-stage, two-sex life table. The results showed that the population parameters of *M. persicae* are higher in three cultivars (Xinong 18, Aiyouku 999, and Aiganyou 558) and lower in Mianxinyou 78 and Zhongshuang 11 compared to Zhongyou 821. This study concluded that *B. napus* cultivars affect the developmental duration, survival rate, and fecundity of *M. persicae* and provide information for choosing a suitable rapeseed cultivar.

## 1. Introduction

*Brassica napus* L., which is a Brassicaceae plant, is the third-largest oil crop in the world according to the FAO data in 2024. The planting area and average yield of oilseed rape has increased in China over the years [1]. Oilseed rape comprises three main varieties: *B. campestris*, *B. napus*, and *B. juncea*, and *B. napus* is the main cultivated species in China due to its exuberant nutritional growth, adaptability, and high yield [2]. However, oilseed rape is infected by different pests, and aphids are identified as the most serious pests affecting oilseed rape in China [1,3,4]. *Myzus persicae* (Sulzer) (Hemiptera: Aphididae), *Lipaphis erysimi* (Kaltenbach) (Hemiptera: Aphididae), and *Brevicoryne brassicae* (Linnaeus) (Hemiptera: Aphididae) were the main aphid species [5]. *Myzus persicae* is a polyphagous pest that damages many economical plants, and its host range includes over 400 plant species [6]. *Myzus persicae* can seriously damage plants through feeding on plant phloem, excreting honeydew, and transmitting plant pathogenic viruses [7,8,9]. The management of *M. persicae* predominantly relies on chemical insecticides. However, the widespread use of insecticides has led to the high resistance of *M. persicae* and negative effects on biodiversity [10]. Consequently, screening aphid-resistant plant cultivars is a viable long-term integrated pest management strategy [11].

Numerous studies indicate that the glucosinolates in plants play an important role in plant–insect interactions [12,13]. The high-glucosinolate content in Brassicaceae leaves has been identified as unsuitable for the growth and reproduction of insects, such as *Plutella xylostella* (Lepidoptera: Plutellidae) [14]. The seeds of *B. napus* have different erucic acid and glucosinolates contents [15,16,17]. China’s national standard verifies that *B. napus* seeds, with an erucic acid content of less than 5% and a glucosinolate content of less than 45 µmol/g in each rapeseed cake, are considered low erucic acid and low glucosinolate content in the *B. napus* variety as food oil [18]. China’s national standard verifies that *B. napus* seeds with an erucic acid content higher than 43% are considered a high-erucic acid variety [19]. Rapeseed oil has high content of erucic acid, which is not easily digested or absorbed by the human body [20,21]. However, high-erucic acid rapeseed is the most important source for producing industrial erucic acid, which is widely used in the field of chemistry [15]. Therefore, in *Brassica napus* seeds, scientists are working to enhance the erucic acid content of seeds to serve industrial materials, while reducing the erucic acid content of seeds to ensure the food oil safety of that produced by rapeseed [20]. However, the impact of *B. napus* cultivars in that their seeds have different glucosinolate contents on the population dynamics of *M. persicae* is still underreported under indoor conditions.

The host plant quality can directly affect the fecundity of herbivorous insects [20]. Population life tables are essential for research on arthropod population ecology, pest management, and host plant resistance [11,22,23]. The age-stage, two-sex life table theory integrates both sexes and various developmental stages of insects, thus providing more detailed population parameters [24,25,26]. And, the age-stage, two-sex life table theory can be used in thelytoky insects, such as *Myzus persicae* [11,24]. This study conducted a comparative analysis of the effects of different *B. napus* cultivars on the survival, developmental duration, and reproduction of *M. persicae* using age-stage, two-sex life tables.

## 2. Materials and Methods

### 2.1. Insects and Plants Cultures

The origin colony of *M. persicae* was reared in the Southwest University of Science and Technology, Mianyang City, Sichuan Province, China. The *Myzus persicae* population was maintained on *B. napus* seedlings in a plastic pot (15 cm diameter) in a 30 × 30 × 30 cm cage, and then housed in an artificial climate incubator (Zhejiang Tupu Yunnong Science and Technology Co., Hangzhou, China), where the climate condition was 25 ± 1 °C, with a photoperiod of 14 L: 10 D and relative humidity of 50 ± 10%. A new and clean 35-day-old *B. napus* plant was added to the insect cage every week. The oldest plants were removed from the cage according to the plant’s condition. The *Myzus persicae* population was adapted on each rapeseed cultivar over two generations before the experiments.

Six *B. napus* cultivars were used in this experiment, and all of them were purchased from a market (Table 1). The high-erucic acid and high-glucosinolate cultivar Zhongyou 821 (ZY821) was used as a control, and the low-erucic acid and low-glucosinolate cultivars were Zhongshuang 11 (ZS11), Mianxingyou 78 (MXY78), Aiganyou 558 (AGY558), Xinong 18 (XN18), and Aiyouku 999 (AYK999). Plastic pots (15 cm diameter × 7 cm height) were used to cultivate the plants using commercial nutrient soil with an organic matter content above 35% and a neutral pH value (Xinluyuan Seedling Substrate Co., Ltd. Liaocheng, China). All plants were grown in an air-conditioned room with a photoperiod of 14 L:10 D, at 25 ± 3 °C, and a relative humidity of 50 ± 10%. All healthy *B. napus* plants with two to five true leaves were used in the subsequent test (about 35 d).

### 2.2. Life Table Study of M. persicae

Thirty-five-millimeter Petri dishes with 0.01 g/L agar medium were prepared. *Brassica napus* leaves were cut and placed adaxial side up on each Petri dish. Ten *M. persicae* adults were put on the backs of the leaves to lay new brown nymphs. After the emergence of new nymphs, at least 30 individuals were selected, and each was maintained in a separate Petri dish separately. The developmental duration, survival number, number of nymphs, and longevity of each individual was recorded every 24 h until death, and a Petri dish with fresh leaves and new agar medium was replaced every two days to ensure food quality and clean environmental conditions during the period. All test individuals were maintained in an artificial climate incubator as previously described. The life tables of *M. persicae* in different *B. napus* cultivars were completed from March to July in 2023. There were 64, 47, 68, 67, 63, and 62 replications in the ZY821, ZS11, XN18, AGY558, AYK999, and MXY78 cultivars, respectively.

### 2.3. Data Analysis

All raw data of *M. persicae* were analyzed by using the TWOSEX-MSChart program and based on the theory of the age-stage, two-sex life table [25,26,27]. The mean value and standard error of each parameter were analyzed using the bootstrap method with 100,000 repetitions, and a paired-bootstrap test was adopted to compare the significance of each parameter (*p* < 0.05) [28,29,30]. All figures were drawn using Sigmaplot 12.5.

In all the data, *x* means age and *j* means the stage of *M. persicae*; *i* indicates age and *y* means the stage of *M. persicae* in the theory condition to calculate life expectancy. The age-stage specific survival rate (*s_xj_*), the age-stage specific fecundity (*f_xj_*), the age-specific survival rate (*l_x_*), the cohort age-specific fecundity (*m*_x_) and the age-specific maternity (*l_x_m_x_*) were calculated. The following formula was used:

(1) $$m_{x}=m\sum_{j=1}^{m}\sum s_{xj}\;f_{xj}/\sum_{j=1}^{m}s_{xj}$$

The population parameters were also calculated, including the intrinsic rate of increase (r), the finite rate of increase (λ), the net reproductive rate (*R*_0_), the mean generation time (T), the mean female fecundity (F), and doubling time (DT) [24]. For age-stage specific life expectancy (e*_xj_*), $S_{iy}^{\prime }$ is the probability that an individual of age *x* and stage *j* would survive to age *i* and stage *y*, assuming that $S_{iy}^{\prime }$ = 1 [24]. The age-stage reproductive value (*v_xj_*) is defined as the contribution of individuals at age *x* and stage *j* to the future population [31]. Finally, the relative fitness (*Rf*) was calculated: *Rf* > 1 shows that the net reproductive rate of the treated groups increased, while *Rf* < 1 shows that the fitness costs of the control groups increased [32]. The parameters were calculated as follows:

(2) $$\sum_{x=0}^{\infty }e^{-r(x+1)}l_{x}m_{x}=1$$

λ = e^r^ (3)

(4) $$R_{0}=\sum_{x=0}^{\infty }l_{x}m_{x}$$

(5) $$F=\frac{\sum_{x=1}^{N_{f}}E_{x}}{N_{f}}$$

*T* = (*lnR*_0_)/r (6)

*DT* = (*In*2)/r (7)

(8) $$e_{xj}=\sum_{i=x}^{n}\sum_{y=j}^{m}S_{iy}^{\prime }$$

(9) $$v_{xj}=\frac{e^{r(x+1)}}{S_{xj}}\sum_{i=x}^{\infty }e^{-r(i+1)}\sum_{y=j}^{m}S_{iy}^{\prime }f_{iy}$$

*Rf* = *R*_0_ value of the treatment groups/*R*_0_ value of the control groups. (10)

## 3. Results

### 3.1. Survival Rate of M. persicae on Six B. napus Cultivars

**Figure 1:**
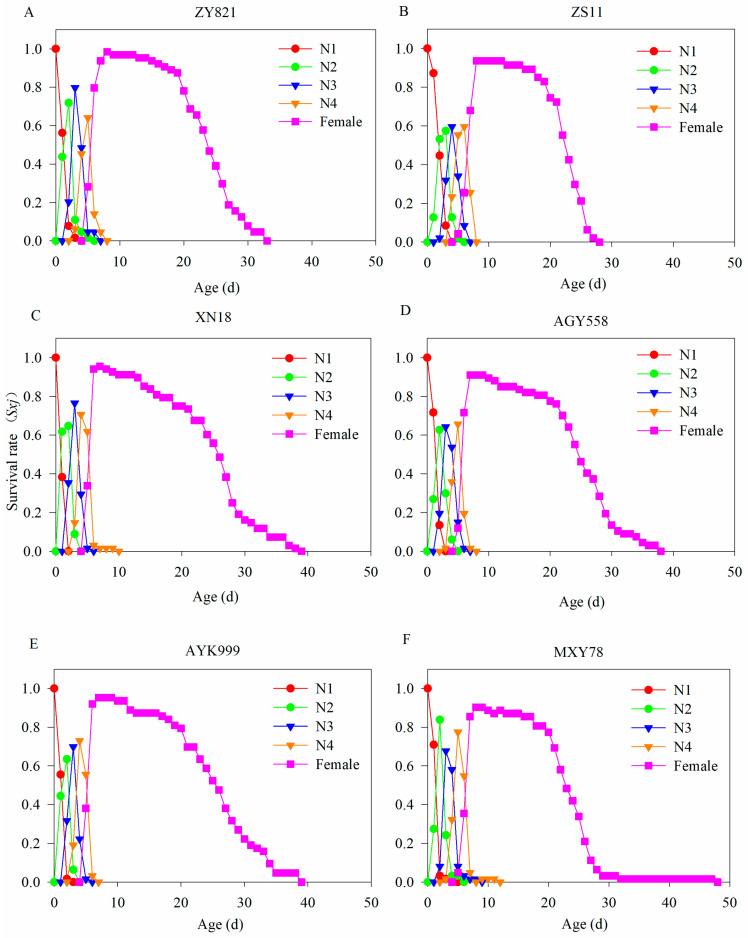
The age-stage survival rate of *M. persicae* feeding on different *B. napus* cultivars. (**A**–**F**) The survival rate of *M. persicae* after feeding on ZY821, ZS11, XN18, AGY558, AYK999, and MXY78, respectively. N1, N2, N3, N4, and females represent the first–fourth instar nymphs and female stage of *M. persicae*.

The age-stage specific survival rates (*S_xj_*) of *M. persicae* from birth to death can be found in six *B. napus* cultivars in Figure 1. *M. persicae*, from first nymphs to adults, was divided into first nymphs, second nymphs, third nymphs, fourth nymphs, and adults. The *M. persicae* were reproduced via parthenogenesis in this study, so we just showed female data. The curves of different instar stages of *M. persicae* overlapped, which was caused by the varied growth rates of *M. persicae* individuals. We found that the survival rate of *M. persicae* at the adult stage was relatively high, although the data from *M. persicae* in MXY78 decreased in the second half of life, but the survival time was longer (Figure 1F).

### 3.2. Developmental Duration of M. persicae on Six B. napus Cultivars

The developmental duration of each stage, female longevity, and the total longevity of *M. persicae* were significantly different on the six *B. napus* cultivars (*p* < 0.05) (Table 2). The developmental duration of *M. persicae* was significantly shorter in XN18 and AYK999 compared to the other four *B. napus* cultivars. Cultivar ZS11 delayed the preadult duration by 1.33 days compared to Cultivar AYK999 (*p* < 0.05). Cultivar AYK999 (20.30 days) survived for a longer time of 4.47 days compared to Cultivar ZS11. Furthermore, the average total longevity of *M. persicae* changed from 21.7 days to 24.81 days when feeding on all *B. napus* species.

### 3.3. The Population Survival Rate and Fecundity of M. persicae in Six B. napus Cultivars

**Figure 2:**
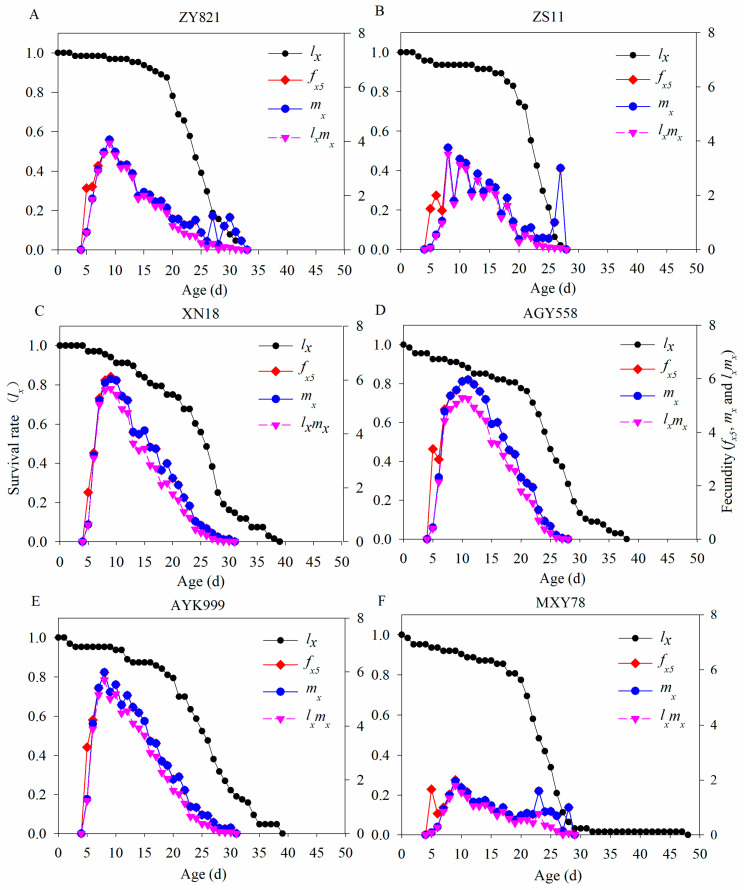
Population survival rate and fecundity of *M. persicae* feeding on different *B. napus* cultivars. *l_x_* represents population survival rate; *f*_x5_ represents female fecundity; *m_x_* represents population fecundity; and *l_x_m_x_* represents population maternity. (**A**–**F**) Population survival rate and fecundity of *M. persicae* after feeding on ZY821, ZS11, XN18, AGY558, AYK999, and MXY78, respectively.

There were significant variations in the population survival rate and fecundity of *M. persicae* in the six *B. napus* cultivars (Figure 2). The population survival rate continuously decreased. An initial increase followed by a decrease in the female fecundity and age-specific reproduction of *M. persicae* occured in six *B. napus* cultivars. Because *M. persicae* are thelytoky insects, the female fecundity and population fecundity partly overlapped in *M. persicae*, since it has thelytoky reproduction (Figure 2). The age-specific fecundity (*m*_x_) had peak values at D9, D8, D9, D11, D8, and D9 on the ZY821, ZS11, XN18, AYG558, AYK999, and MXY78 Cultivars, respectively. The highest *m*_x_ values were at 4.07, 3.75, 6.03, 5.97, 5.98, and 1.96 on ZY821, ZS11, XN18, AYG558, AYK999, and MXY78, respectively (Figure 2).

### 3.4. The Population Parameters of M. persicae in Six B. napus Cultivars

The population parameters of *M. persicae* showed significant differences in the six *B. napus* cultivars (Table 3). The parameters of *M. persicae* in AYK999 were higher than those observed in those feeding on five other *B. napus* cultivars. The mean generation time of *M. persicae* was shorter at 10.47 days in AYK999, and it produced a 1.19-fold delay in MXY78 comapred to that in AYK999. The intrinsic rate of increase (0.40), net reproductive rate (63.43), the finite rate of increase (1.49), and *Rf* value (1.60) were the largest, and the double time (1.75) was the lowest in AYK999. Oppositely, the parameters of *M. persicae* in MXY78 were relatively lower, and the double time (3.04) of *M. persicae* in MXY78 was the longest.

Since the intrinsic rate of increase can accurately and comprehensively reflect the data on insect population growth, reproduction, and survival rate, it is used as an indicator to compare population growth and an important parameter to identify breed resistance. Considering all parameters, MXY78 had the lowest r value, indicating that the MXY78 variety is the most resistant to aphids. The r value of AYK999 was the highest, so it was the least resistant to *M. persicae*.

### 3.5. Life Expectancy of M. persicae in Six B. napus Cultivars

**Figure 3:**
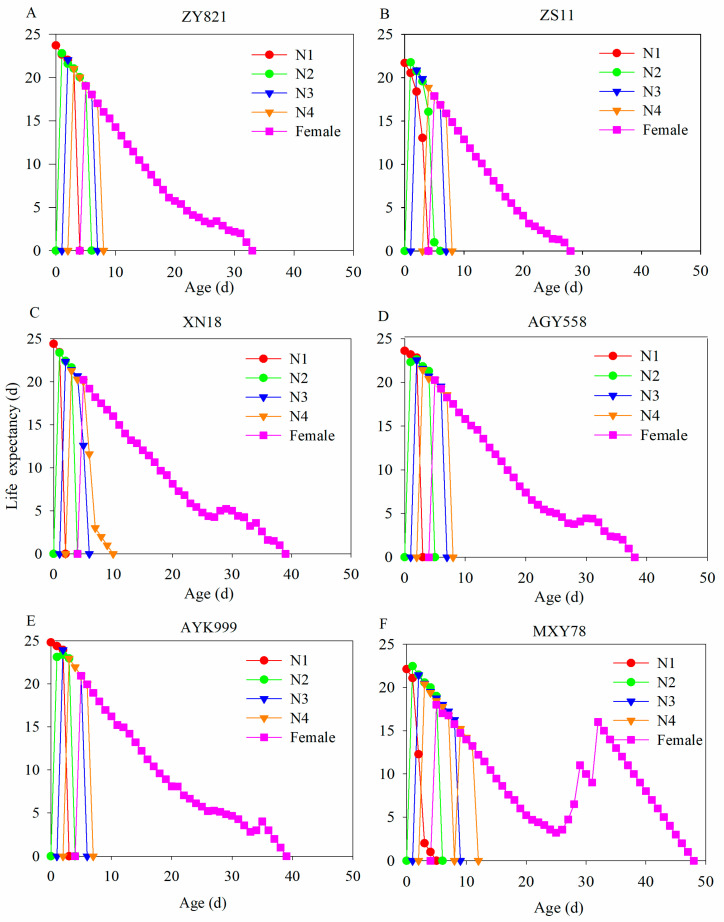
Life expectancy of *M. persicae* in each developmental stage feeding on different *B. napus* cultivars. (**A**–**F**) The life expectancy of *M. persicae* after feeding on ZY821, ZS11, XN18, AGY558, AYK999, and MXY78, respectively. N1, N2, N3, N4, and female represent first–fourth instar nymphs and female stages of *M. persicae*.

The life expectancy of *M. persicae* in six *B. napus* cultivars was the greatest at age 0. The highest e*_xj_* values at age 0 for *M. persicae* feeding on ZY821, ZS11, XN18, AGY558, AYK999, and MXY78 were 23.70, 21.77, 24.40, 23.61, 24.81, and 22.11 days, respectively (Figure 3). Then, the life expectancy of *M. persicae* at each stage gradually decreased with the prolonged aphid lifespan.

### 3.6. Reproductive Value of M. persicae in Six B. napus Cultivars

**Figure 4:**
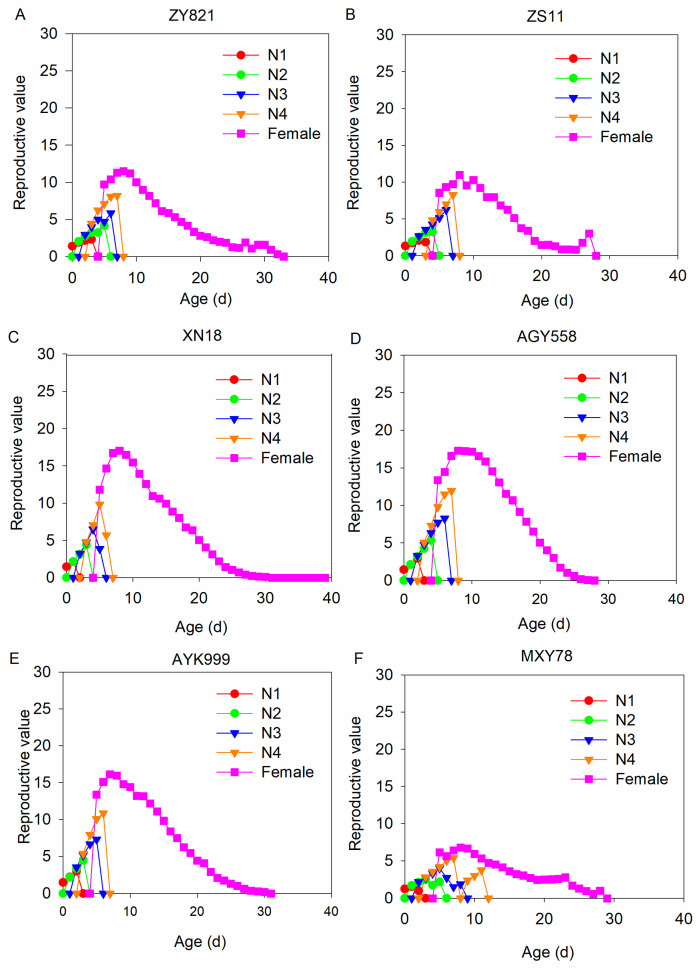
Reproductive value of *M. persicae* in each developmental stage feeding on different *B. napus* cultivars. (**A**–**F**) The reproductive value of *M. persicae* after feeding on ZY821, ZS11, XN18, AGY558, AYK999, and MXY78, respectively. N1, N2, N3, N4, and female represent first–fourth instar nymphs and female stages of *M. persicae*.

The reproductive value of *M. persicae* at each stage was gradually increased and finally declined as the lifespan increased in the six *B. napus* cultivars (Figure 4). The female adult stage of *M. persicae* had the maximum contribution in all populations numbers.

### 3.7. Principal Component Analysis of Different Brassica napus Seeds and Developmental Duration and Fecundity of Other M. persicae in Six B. napus Cultivars

**Figure 5:**
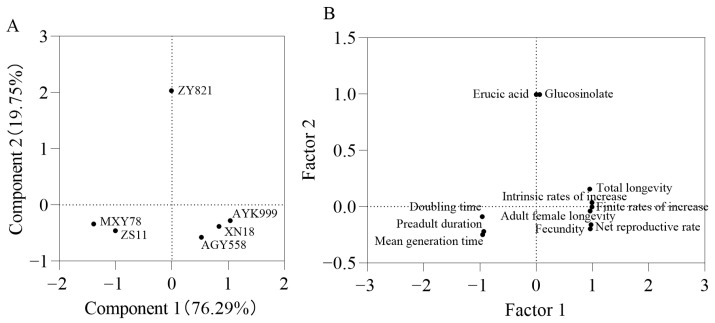
Principal component analysis (PCA) for different *B. napus* cultivars based on two substance contents in *Brassica napus* seeds and nine *M. persicae* population indexes. (**A**) PC scores of six rapeseed cultivars. (**B**) Loading plot was each index of *M. persicae* and erucic acid and glucosinolate content in six *B. napus* seeds. The factors included erucic acid: erucic acid content (%), glucosinolate: glucosinolate (µmol/g/rapeseed cake), doubling time, preadult duration (d), mean generation time (d), total longevity (d), intrinsic rates of increase (d), finite rates of increase (d), adult female longevity (d), fecundity (d), and net reproductive rate.

The erucic acid and glucosinolate content in six *B. napus* seeds and the *M. persicae* individual and population indexes in six *B. napus* seedlings were analyzed via PCA (Figure 5). PCA revealed that the data could be divided into two principal components (PCs): the first PC accounted for 76.29% and the second PC accounted for 19.75% of the variation. The cultivars (AYK999, AGY558, and XN18) were clusterd, and MXY78 and ZS11 were clearly separated from ZY821 and other plant cultivars (Figure 5A).

Meanwhile, the doubling time, preadult duration, and mean generation time showed a strong negative correlation. Conversely, the total longevity, intrinsic rates of increase, finite rates of increase, adult female longevity, fecundity, and net reproductive rate exhibited a strong positive correlation (Figure 5B).

## 4. Discussion

*Brassica napus* is one of the most important crops in China. However, the occurrence of pests and diseases has increased in *B. napus* fields [1]. In the study, *M. persicae* showed higher fecundity and intrinsic rates of increase on three double-low *B. napus* cultivars (XN18, AGY558, and AYK999) compared to the double-high *B. napus* cultivar ZY821. In contrast, a weaker fecundity and lower intrinsic rate of increase were observed in ZS11 and MXY78. Different *B. napus* cultivars significantly affected the population parameters of *M. persicae*. These results were consistent with a previous report. Li et al. (2023) have reported that *B. napus* varieties can have high or low resistance to aphids [33].

The host plants can affect the survival rate, developmental duration, and fecundity of many insects [11,14,34]. Glucosinolates are vital in secondary metabolites, found in cruciferous crops, and have defensive functions in plant–insect interactions [33]. When plant tissue is damaged, such as by a chewing insect or through mechanical injury, the intact glucosinolates stored in the vacuole come into contact with the enzyme myrosinase, leading to the production of isothiocyanate, nitrile, and oxazolidinethione [10,11]. Glucosinolates are favored by the chewing–feeding insects Lepidoptera for feeding and oviposition, but do not contribute to expanding phloem-feeding insects [13,35]. Evidence showed that the glucosinolate levels of different *Arabidopsis* genotypes affect host plant suitability and insect performance for generalist and specialist herbivores, and that the number of aphids was negatively related to the constitutive glucosinolate content of the plant [36]. The generalist aphid *M. persicae* and specialist aphid *B. brassicae* infested the plants and significantly increased the glucosinolate levels in *Arabidopsis*. However, direct evidence regarding how the glucosinolate content of plants affects the growth and development of aphids remains relatively scarce [37].

The seeds of *B. napus* have different glucosinolate contents [13]. Can different *B. napus* cultivars with seeds have different glucosinolate contents and affect aphid resistance? One previous article discovered the relationship between bird damage and the glucosinolate content of seeds in *B. napus*, where the bird damage increased in *B. napus* plants with a glucosinolate content of lower than 23 µmol/g in the seed stage [37]. Li (2006) suggests that increasing the glucosinolate content in *B. napus* leaves could enhance the resistance of the plant to disease and pests [38]. The aphid resistance of rapeseed in our results was consistent with the point of Mewis et al. [35]. Nonetheless, it is essential to acknowledge that various factors like host plant species, nitrogen contents, temperature, water, and soil conditions influence insect population dynamics in the field [39,40,41]. Therefore, further field trials are needed to comprehensively compare aphid resistance among different *B. napus* cultivars.

Our current study in laboratory conditions lays the groundwork for more advanced research to assess practical applications in real and complex field environments. Under field conditions, multiple trophic interactions and the attack sequence of insects can significantly influence plant–aphid interactions. In the agricultural ecosystem, growing different *B. napus* cultivars can naturally suppress *M. persicae* populations, reducing reliance on chemical pesticides. Integrating cultivar selection into pest management strategies can achieve sustainable crop protection while supporting ecological balance, and promote the development of organic farming [42].

## 5. Conclusions

This study found that six *B. napus* cultivars affected the population of *M. persicae* based on the age-stage, two-sex life table method. Cultivars ZS11 and MXY78 were the least suitable hosts, as evidenced by the lowest net reproductive rate and highest mean generation time. So, two *B. napus* cultivars (ZS11 and MXY78) were unfavorable for raising the *M. persicae* numbers. Three *B. napus* cultivars (XN18, AGY558, and AYK999) in the seed stage with a low glucosinolate content posed a higher risk of increasing the *M. persicae* population. Due to the erucic acid content and glucosinolate content of seeds varying in *B. napus*, choosing the suitable rapeseed cultivar is important for pest management, and we should to pay more attention to the pest management strategies of double-low rapeseed cultivars in agriculture ecosystems.

## Figures and Tables

**Table 1:** Information of erucic acid, glucosinolate, and oil content in different *B. napus* seeds on commercial package.

Cultivar Name	Abbreviation Name	Erucic Acid Content (%)	Oil Content (%)	Glucosinolate (µmol/g/ Rapeseed Cake)
‘Zhongyou 821’	ZY821	45.45%	_	113.5
‘Zhongshuang 11’	ZS11	0	49.04%	18.84
‘Xinong 18’	XN18	0	41.20%	23.19
‘Aiganyou 558’	AGY558	0.2%	49.23%	21.07
‘Aiyouku 999’	AYK999	1.08%	41.04%	28.04
‘Mianxingyou 78’	MXY78	0.4%	46.18%	20.31

Note: All erucic acid, glucosinolate, and oil contents in *B. napus* seeds from the commercial package. The part information (ZS11, MXY78, XN18, and AYK999) also can be found on website (http://www.zys.moa.gov.cn/, accessed on 7 July 2025), but the information of ZY821 and Aiganyou 558 cultivar cannot be found on this website.

**Table 2:** Developmental duration and longevity of *M. persicae* feeding on different *B. napus* cultivars.

Duration (d)	ZY821	ZS11	XN18	AGY558	AYK999	MXY78
1st-instar duration/d	1.66 ± 0.08 bc	2.36 ± 0.12 a	1.38 ± 0.06 d	1.86 ± 0.08 b	1.57 ± 0.07 c	1.72 ± 0.06 bc
2nd-instar duration/d	1.33 ± 0.06 ab	1.41 ± 0.08 ab	1.35 ± 0.06 ab	1.30 ± 0.06 bc	1.15 ± 0.05 c	1.52 ± 0.08 a
3rd-instar duration/d	1.60 ± 0.07 a	1.45 ± 0.08 ab	1.43 ± 0.06 ab	1.60 ± 0.07 a	1.32 ± 0.07 b	1.59 ± 0.08 a
4th-instar duration/d	1.37 ± 0.06 cd	1.75 ± 0.11 ab	1.52 ± 0.07 bc	1.32 ± 0.06 d	1.58 ± 0.06 b	1.88 ± 0.07 a
Preadult duration/d	5.95 ± 0.10 b	6.95 ± 0.13 a	5.66 ± 0.06 c	6.11 ± 0.08 b	5.63 ± 0.87 c	6.71 ± 0.13 a
Adult female longevity/d	18.10 ± 0.59 bc	15.93 ± 0.47 d	19.55 ± 0.86 ab	19.16 ± 0.82 ab	20.30 ± 0.06 a	16.76 ± 0.68 cd
Total longevity/d	23.72 ± 0.67 ab	21.70 ± 0.79 b	24.40 ± 0.95 a	23.61 ± 1.04 ab	24.81 ± 1.04 a	22.11 ± 0.96 ab

Note: Data are represented as mean ± SE and analyzed using TWOSEX-MSChart program. Different letters in the same row indicate significant differences at *p* < 0.05, considering the paired-bootstrap test.

**Table 3:** Populational parameters of *M. persicae* feeding on different *B. napus* cultivars.

Parameters	ZY821	ZS11	XN18	AGY558	AYK999	MXY78
Intrinsic rates of increase (r)/d^−1^	0.34 ± 0.005 c	0.28 ± 0.006 d	0.39 ± 0.006 ab	0.37 ± 0.007 b	0.40 ± 0.006 a	0.23 ± 0.007 e
Finite rates of increase (λ)/d^−1^	1.40 ± 0.008 c	1.33 ± 0.008 d	1.47 ± 0.009 ab	1.45 ± 0.010 b	1.49 ± 0.008 a	1.26 ± 0.008 e
Net reproductive rate (*R*_0_)	39.69 ± 1.83 b	29.04 ± 1.67 c	62.16 ± 3.14 a	63.19 ± 3.57 a	63.43 ± 2.99 a	17.19 ± 1.33 d
Mean generation time/d	10.92 ± 0.17 bc	11.97 ± 0.20 a	10.71 ± 0.15 cd	11.22 ± 0.14 b	10.47 ± 0.13 d	12.47 ± 0.25 a
Fecundity	40.32 ± 1.74 b	31.02 ± 1.33 c	65.03 ± 2.80 a	68.29 ± 3.04 a	66.60 ± 2.52 a	18.38 ± 1.28 d
Doubling time (DT)	2.06	2.46	1.80	1.88	1.75	3.04
Relative fitness (*Rf*)	1	0.73	1.57	1.59	1.60	0.43

Note: Data are mean ± SE and analyzed using TWOSEX-MSChart program. Different letters in the same row indicate significant differences at *p* < 0.05, considering the paired-bootstrap test.

## Data Availability

The raw data supporting the conclusions of this article will be made available by the authors on request.
